# Recent Advances on the Regulations of Organic Anion Transporters

**DOI:** 10.3390/pharmaceutics16111355

**Published:** 2024-10-24

**Authors:** Zhou Yu, Guofeng You

**Affiliations:** Department of Pharmaceutics, Rutgers, The State University of New Jersey, 160 Frelinghuysen Road, Piscataway, NJ 08854, USA; zy185@pharmacy.rutgers.edu

**Keywords:** drug transporter, organic anion transporter, regulation, post-translational modification, diseases, drug disposition

## Abstract

The organic anion transporter (OAT) family of over 10 members within the solute carrier (SLC) superfamily of membrane proteins plays critical roles in facilitating the flux of negatively charged molecules in and out of cell membranes. These anionic molecules include various endogenous and exogenous compounds such as signaling molecules, nutrients, metabolites, toxins, and drugs. Therefore, OATs actively contribute to the systemic homeostasis and efficacy of therapeutics. This article provides a brief overview on recent advances in the understanding of the regulatory mechanisms that control the expression and activity of OATs in both health and diseases.

## 1. Introduction

The organic anion transporter (OAT) family of over 10 members within the solute carrier (SLC) superfamily of membrane proteins plays critical roles in facilitating the flux of negatively charged molecules in and out of cell membranes. These anionic molecules include various endogenous and exogenous compounds such as signaling molecules, nutrients, metabolites, toxins, and drugs. OATs are expressed at the physiological membranes of various tissues, including the kidney, brain, placenta, liver, and intestines. These transporters are not only essential from the aspect of human physiology and pathophysiology but also essential from the aspect of drug absorption, distribution, metabolism, and elimination (ADME) [1,2,3,4,5,6]. As a result, a great deal of effort has been put into understanding the mechanisms that underlie the regulation of OATs. In the current review, we present the recent progress in discovering the regulations of OATs at the genetic level and post-translational level, as well as various factors involved in such regulations.

### 1.1. General Characteristics of OATs

The members of the OAT family share some common structural features. They are membrane proteins with 12 transmembrane domains (TMDs), a large extracellular loop between TMD1/2, and a large intracellular loop between TMD6/7. Both the amino terminus and the carboxyl terminus are localized intracellularly. Multiple glycosylation sites reside in the large extracellular loop between TMD1/2. The glycosylation of OATs is critical for the transporters to bring traffic to the cell surface after synthesis. The large intracellular loop between TMD6/7 and both N- and C-termini contain multiple potential sites for the regulation of the transporters such as those sites for phosphorylation, ubiquitination, and SUMOylation. An important functional feature of OATs is that they recognize a wide range of substrates including endogenous substances, their metabolites, and xenobiotic molecules such as toxins from the environment and therapeutic drugs, which make them essential players in the homeostasis and pharmacological responses of the body [1,2,3,4,5,6].

### 1.2. Interaction of OATs with Endogenous and Exogenous Substances

Although the members of OATs are expressed in multiple tissues, the majority of studies focused on OATs expressed in the kidney. One of the vital functions of the kidney is the removal of undesirable substances from the blood followed by their elimination via urine. Much of the removal/elimination of the organic anions such as nutrients, metabolites, therapeutics, and toxins are accomplished by the concerted action of transporters expressed at the blood-facing basolateral membrane and urine-facing apical membranes of the kidney proximal tubule cells. Transporters at the basolateral membrane move the organic anions from the blood into the proximal tubule cells. Once inside cells, these substances then exit into the urine by moving across the apical membrane via other transporters such as the multidrug resistance-associated proteins. In humans, OATs 1, 2, 3, 4, 10, and URAT1 are detected in the kidney, whereas in rodents, the expression of some OATs are different from those in humans, which may explain some of the functional differences between these species. OATs 1, 2, and 3 are expressed at the blood-facing basolateral membrane of the kidney proximal tubule cells in humans. OAT4 and URAT1 (another membrane of the OAT family) are expressed at the urine-facing apical membrane of the proximal tubule cells. OAT4 reabsorbs anionic substrates from urine back into the tubule cells. OAT10 transports nicotine and uric acid. URAT1 mediates the reabsorption of urate through monocarboxylate exchange [1,2,3,4,5,6].

Numerous endogenous substances and their metabolites are removed from the body by renal OATs in keeping with the body’s homeostasis. More than 100 metabolites were altered in the plasma of Oat1 and Oat3 knockout mice (OAT1KO, OAT3KO) [7,8,9], among which many are endogenous substrates of OATs, such as bile acids, indoxyl sulfate, and various signaling molecules. Thus, the abnormal expression and function of OATs under certain pathophysiological conditions may influence the control of these endogenous substances and their metabolites.

In addition, to interact with numerous endogenous substrates, OATs also handle a large variety of exogenous substances such as toxins from the environment and therapeutics. When multiple drugs are taken together, these drugs may mutually impact each other’s pharmacokinetics via competing with the same transporters, therefore causing drug–drug interaction. Such drug–drug interaction can be either beneficial or detrimental. For example, probenecid, a classic inhibitor for OATs, are used clinically with other OAT substrate drugs to extend their duration in the body, thereby enhancing their therapeutic outcome. Probenecid was given to patients for reducing the renal clearance of penicillin, an OAT substrate, so the antimicrobial effects of penicillin can be enhanced under the same dose [10]. On the other hand, the co-administration of mizoribine and bezafibrate enhanced the build-up of bezafibrate in circulation, increasing the risk of advancing rhabdomyolysis [11].

Since OATs recognize a large variety of substrates, both endogenous and exogenous, drug–drug interaction, drug–toxin interaction, and drug–endogenous substrate interaction must be taken into consideration clinically.

## 2. OATs in Kidney Injury and Diseases

The connections between OATs and various diseases have been reported through animal studies, as well as clinical observations. These reported cases are often very complex and difficult to interpret. For instance, certain disease states could lead to the altered expression, function, and localization of OATs. Such altered OATs could then change the physiological functions of various organs, resulting in new diseases or the progression of existing diseases [2,6,12].

### 2.1. Acute Kidney Injury (AKI)

Acute kidney injury (AKI) is a pathological condition exhibiting a sudden decrease in renal functions. The current known causes of clinical AKI are renal ischemia/reperfusion, physical kidney injury, and toxin/drug-induced renal toxicity [2,6]. Renal ischemia/reperfusion often reduces the glomerular filtration rate (GFR) and renal tubular functions such as secretion and reabsorption, leading to the accumulation of uremic toxins [6,13]. In a rat model of ischemia, the Sauvant group showed that the mRNA and protein expression of rOat1 and rOat3 were significantly decreased in the kidneys [13,14]. In another rat model of ischemic AKI, Saito et al. showed a reduced expression of rOat1 and rOat3 and an accumulation of indoxyl sulfate, an OAT substrate. Such perturbation could be reversed by several clinically used anti-inflammatory drugs such as meclofenamate, quercetin, and resveratrol [15,16]. Ischemia-induced AKI down-regulated the expression and function of rOat1 and rOat3 via the activation of prostaglandin E receptor 4 in a rat model of ischemia/reperfusion AKI [14,17].

Numerous toxins and therapeutic agents are also able to induce AKI, such as aminoglycosides antibiotics and angiotensin-converting enzyme inhibitors [2,3,6]. In a rat model of gentamicin-induced AKI, the mRNA and protein expressions of rOat1 and rOat3 were significantly reduced, resulting in defective renal functions and the accumulation of metabolites and uremic toxins [18]. In a rat model of vancomycin-induced nephrotoxicity, the expression levels of rOat1 and rOat3, as well as rOct2 and P-gp, were significantly reduced. The following treatment with rhein reversed such decreases and enhanced the excretion of blood urea nitrogen and creatinine. The authors attributed the protective effects of rhein to the activation of nuclear factor erythroid 2-related factor 2 (Nrf2) and the inhibition of renal cell apoptosis [19]. In a rat model of cisplatin-induced AKI, the expression and function of rOat1 in the kidney were greatly decreased. The following treatment in AKI rats using hyperoside, an anti-oxidative flavonol glycoside compound isolated from herbal plants, reduced kidney damage and enhanced renal excretion via up-regulating the expression and function of renal rOat1. The authors revealed that the activation of HIF-1α and pregnane X receptor (PXR) by rhein led to the up-regulation of rOat1 [20]. In addition, in animal studies on heavy metal-induced renal toxicity, the uptake transporters Oat1, Oat3, and Oct2 exhibited decreased expression levels, while the expressions of efflux transporters such as Mdr1 and Mrp2/4 were significantly increased. The authors indicated that such a decreased uptake and enhanced clearance of heavy metals functioned as protection against heavy metal accumulation and related toxicity in the kidney [21,22]. Interestingly, OATs could also contribute to AKI progression since their substrate drugs may damage kidney cells once transported in. For example, methotrexate can be transported into kidney cells by OATs and causes kidney injury. Resveratrol, an anti-inflammatory drug, directly inhibited rOat1 and rOat3, and significantly decreased the OAT-mediated renal uptake of methotrexate, leading to less methotrexate accumulation in renal cells and reduced renal toxicity [23].

### 2.2. Chronic Kidney Failure (CKF)

Chronic kidney failure gradually and continuously reduces the glomerular filtration rate and renal clearance, leading to the gradual accumulation of metabolites, uremic toxins, and clinical drugs in the kidneys as well as other organs across the whole body [2,12,24]. During this process, the increased metabolites and uremic toxins can also modulate the expression and function of OATs. For example, in a rat model of CKF, the mRNA and protein expression of rOat1, rOat2, and rOat3 were all significantly decreased. Furthermore, serum extracted from the above CKF rats reduced the expression of human OATs in cultured human renal proximal tubule cells [24]. This study indicated that the elevated metabolites and uremic toxins in the CKF rats were responsible for the observed down-regulation of rat Oats and human OATs [2,24]. In the adenine-induced rat model of CKF, the mRNA and protein expression levels of rOat1 and rOat3 were significantly down-regulated [25]. P-cresyl sulfate, one typical uremic toxin, decreased rOat1 expression in an adenine-induced model of CKF. In these adenine-induced CKF rats, the protein expression of rOat1 was reduced by 40% after a 5-week intake of p-cresyl sulfate [26]. Kong et al. showed, in rat model with CKF, that the elevated uremic toxins, indoxyl sulfate, and hippuric acid significantly inhibited the renal clearance of morinidazole metabolites via inhibiting the rOat-mediated drug elimination, since morinidazole metabolites are substrates of rOat1 and rOat3 [27,28].

The introduction of mOat1 and mOat3 knockout mice also shed light on the complicated relationship between uremic toxins and OATs [7,8]. In mOat1 knockout mice, the plasma concentrations of indoxyl sulfate, kynurenine, and xanthurenic acid were all elevated. Also, these uremic toxins were proven to inhibit mOat1 function in cell culture [9]. Similarly, in mOat3 knockout mice, the plasma levels of p-cresyl sulfate and indole acetate were significantly increased. In vitro experiments confirmed their inhibitory effects on mOat3 [7]. In kidney transplant patients, the plasma levels of several uremic toxins were significantly elevated after administering drugs that inhibited OAT1 or OAT3 [29]. In summary, the interactions between uremic toxins and OATs are very complex in CKF conditions. The accumulated metabolites and uremic toxins from reduced renal functions in CKF patients could in turn reduce OAT function further, forming a negative feedback and worsening the conditions caused by CKF [2,8].

Recently, the potential connections between OAT single-nucleotide polymorphism (SNP) and clinical diseases were investigated to explore the physiological/pathological roles of OATs in patients. For instance, the comparison between normal subjects and patients revealed that patients with chronic kidney disease (CKD) showed a higher rate of the −475 SNP in the 5′ regulatory region of the OAT1 gene than normal subjects [30]. Furthermore, the −475 SNP with T to G transversion decreased the hepatoma-derived growth factor (a known transcription repressor) binding to the OAT1 gene. Taken together, these findings suggested that the −475 SNP of OAT1 may up-regulate OAT1 expression and increase the renal uptake of OAT1 substrates (including toxins and drugs), resulting in more nephrotoxicity and eventually chronic kidney failure [30].

### 2.3. Hyperuricemia

Hyperuricemia is a disease with high urate concentrations in the blood and tissues. Gout, caused by hyperuricemia, is a type of arthritis that causes sudden pain and swelling in the joints. Hyperuricemia also leads to uric acid nephropathy, which is a kidney disease related to proteinuria and hypertension [2,6,31]. The excretion and reabsorption of urate mainly depend on the kidney to achieve whole-body urate homeostasis. In the kidney, urate excretion is mediated by OAT1, OAT3, ABCG2, and NPT1, while URAT1, OAT4, and GLUT9 mediate the reabsorption of urate. Thus, expression and functional changes in the excretion and/or reabsorption transporters would result in hyperuricemia [31]. Lower mRNA and protein expressions of OAT1 and OAT3 were consistently reported in animal studies on uric acid nephropathy [32,33,34]. In a rat model of hyperuricemia, the rats orally treated with anserine showed reduced blood urea nitrogen and creatinine levels compared to the untreated group. Upon further investigation, the expression of rOat1 and rOat3 were up-regulated by anserine (increased urate excretion). In contrast, rUrat1 expression was down-regulated simultaneously (decreased urate reabsorption), indicating that anserine’s relieving effects on hyperuricemia were via regulating rOat1, rOat3, and rUrat1 [35]. Furthermore, based on the gene analysis of OAT4 in healthy subjects and gout patients, an SNP (position at chromosome 11: 64088038, A/G) on OAT4 was involved in the renal underexcretion-type gout. The authors indicated that genetic/functional changes in OAT4 in renal proximal tubule cells would alter urate reabsorption and lead to gout [36,37]. Through bioinformatic comparisons between normal subjects and hyperuricemia patients, three SNPs in the human URAT1 gene were discovered to be significantly associated with hyperuricemia, while two other SNPs of URAT1 were positively correlated with hypouricemia, suggesting the critical role of URAT1 in urate handling [38].

### 2.4. Obesity and Diabetes

The expressions and functions of OATs under obese or diabetic conditions were investigated in various animal models. In a streptozotocin-induced rat model of diabetes, the protein expression and transport activity of rOat3 were significantly reduced. Such reductions were reversed following insulin injection [39]. In another model for diabetes using Ins2Akita mice, the mRNA and protein expression of mOat1, mOat2, and mOat3 were all decreased [40]. In obese rats fed with a high-fat diet, the transport activity and protein expression of renal rOat3 were both decreased significantly. Treatment with atorvastatin or vildagliptin in those obese rats partially restored the reduced rOat3 transport activity [41]. Furthermore, in an obese rat model showing renal inflammation and reduced rOat3 expression, administering fructooligosaccharides inhibited obesity-induced inflammation, fibrosis, and apoptosis. It also reversed the reduced expression and transport activity of rOat3 [42]. In addition to the abnormal expression of rOat1/3 in rat models, human OAT2 protein expression was significantly up-regulated in the liver of obese and steatosis patients [43,44]. Human OAT7 protein expression was also significantly increased in obese subjects compared to normal subjects [43,44].

### 2.5. Cancer

Multiple types of cancers, as well as their treatment approaches, could significantly affect the expression and transport function of OATs. For instance, OAT2 expression was elevated in the tumor tissue of metastatic colorectal cancer after the chemo treatment of FOLFOX. Such higher OAT2 expression was positively associated with better tumor response, which might serve as a good predictor for the outcome of FOLFOX treatment. The authors indicated the involvement of OAT2 in the uptake of FOLFOX drugs as a potential explanation for the correlation [45]. In hepatocellular carcinoma (HCC) patients who were treated with local ablation therapy, patients with lower OAT2 expression in the liver exhibited increased rates of multifocal recurrence than those having a normal expression level of OAT2. Furthermore, such reduced OAT2 expression was significantly associated with potential HCC occurrence in patients with the hepatitis C virus. The authors suggested that reduced OAT2 expression might induce hepatic carcinogenesis via increasing orotic acid concentration in nearby tissues and promoting oxidative stress and mitochondrial dysfunction in hepatocytes [46]. More recently, an advanced analysis of multiple RNA-Seq datasets from The Cancer Genome Atlas indicated that the expressions of OAT1, OAT2, OAT3, and URAT1 were significantly decreased in the tumor kidney tissues from cancer patients when compared to normal kidney tissues. These cancer patients with lower OAT expression also tended to have a shorter overall survival time. Those observations and correlations suggested that the reduced expression/function of OATs could have systemic effects on both tumor progression (e.g., via disrupted metabolites and cell signaling) and treatment responses (e.g., via altered excretion of chemotherapeutic agents) [47].

### 2.6. Liver Diseases

Cholestasis is a liver disease caused by bile duct obstruction or impaired liver function where the bile flow out of the liver is obstructed or significantly reduced. Several studies have revealed the altered expression of OATs in animal models of cholestasis. For example, in the rat model of bile duct ligation (BDL)-induced cholestasis, rOat1 protein expression was significantly reduced, while the expression of rOat3 was elevated [48]. In another rat model of alpha-naphthyl isothiocyanate (ANIT)-induced cholestasis, the protein expression and functions of rOat1 and rOat3 were both decreased. The authors postulated that the elevated bile acids and bilirubin from cholestasis could activate PKC and lead to the increased internalization and degradation of rOat1 [49]. The variation in rOat3 expression in these reports was likely due to the different approaches to generating cholestasis models (BDL-induced vs. ANIT-induced biliary obstruction). Furthermore, one systemic study investigating the changes in renal transporter expressions in patients of various chronic liver diseases revealed that the protein expression of OAT3 was significantly decreased in NASH (nonalcoholic steatohepatitis), ALD (alcohol-associated liver disease), HCV (viral hepatitis C), and combined ALD/HCV. The expression of OAT4 was also reduced in the kidneys of NASH patients. Also, the expression of URAT1 showed a decreasing trend in the ALD and ALD/HCV patient samples. Interestingly, only OAT2 exhibited a significantly increased expression in NAFLD (nonalcoholic fatty liver disease) patients. The authors emphasized the clinical relationship between OATs and chronic liver diseases in predicting and preventing drug–drug interactions and adverse drug responses in such patients [50].

Other diseases could also affect the expression of OATs. In a rat model of viral infection and inflammation using poly I:C, the mRNA and protein expressions of rOat1, rOat2, rOat3, and rUrat1 were notably down-regulated in rat kidneys, indicating the potential changes in transporter-mediated drug dispositions under infection and inflammation conditions [51]. In another study using patient placenta samples, the mRNA expression of OAT4 was significantly decreased in placenta samples from HIV(+) patients compared to those from HIV(−) subjects, suggesting the transporter-mediated placental functions could be altered or disrupted during HIV infection [52].

Obviously, the possible alterations in OAT expressions and functions under disease conditions should be carefully assessed for adjusting drug dosage to achieve an effective and successful therapy. Therefore, further studies on the mechanisms underlying the changes in OAT expression and function in various diseases are of great value in improving the therapeutic efficacy of drugs and reducing toxicity in clinical uses.

## 3. Regulations of OATs

OATs contribute significantly to many physiological and pathological processes and to the therapeutic outcome of numerous drugs. Therefore, understanding the regulatory mechanisms governing OAT function has profound significance. OAT expression and activity can be regulated at various levels, such as at the levels of transcription, translation, and post-translation.

### 3.1. Regulation of OATs by Transcriptional and Translational Control

#### 3.1.1. Epigenetic Regulation

Epigenetic regulations, such as DNA methylation and histone acetylation, are critical mechanisms for gene regulation. They are able to regulate target gene expression without changing its DNA sequence. DNA methylation is the attachment of methyl groups to a DNA molecule, and these predominantly occur in the promoter region of the gene, resulting in the inhibition of gene transcription [53,54,55,56]. Histone acetylation mostly happens at lysine residues at the N-terminus of histones located around the transcription start site of a gene. The addition of acetyl groups to histones would increase the accessibility of DNA chains for replication and transcription, leading to the activation of target genes [53,54,55,56].

Previous studies have revealed that the expression of several OATs can be regulated by epigenetic modifications. It was found that the promoter activity of OAT1 and OAT3 can be repressed by increased DNA methylation in the promoter region. Also, the tissue-specific expressions of OAT1 and OAT3 were attributed to the varied DNA methylation around the transcription start sites [57,58]. In a recent study, Wang et al. discovered that a lower level of histone acetylation was involved in the transcriptional repression of OAT2 in human hepatocellular carcinoma [59]. Furthermore, the inhibition of HDAC4, one of the classic histone deacetylases, was found to be a critical factor in promoting histone acetylation and increasing the mRNA and protein expression of mouse Oat1 in a mouse model of nociceptive hypersensitivity [60].

#### 3.1.2. Nuclear Receptors

Nuclear receptors (NRs) are one of the major players in the gene regulation of transporters. They are cellular proteins working as DNA transcription factors. After direct binding with both endogenous and exogenous small molecule ligands, NRs will translocate to the cell nucleus and bind to specific sites around the promoter regions to modulate the transcription of target genes. Generally, the activation of NRs will lead to a significantly altered expression and function of various transporters [53,54,55,56].

Several NRs have been shown to be involved in the regulation of OATs. It has been established that the promoter activity of OAT1 and OAT3 was significantly up-regulated by the activation of hepatocyte nuclear factor (HNF)-1α and HNF-4α [53,58,61]. B cell CLL/lymphoma 6 (BCL6) is a transcription factor that increased the expression of rat Oat1 and Oat3 and enhanced the promoter activity of human OAT1 in cell culture [62,63]. The activation of liver X receptors (LXRs) decreased the expression of human OAT1 in an OAT1-overexpressing cell model, as well as mouse Oat1 in the kidney of mice [64]. BMAL1 (basic helix–loop–helix ARNT-like 1) acts as a transcription factor and is a central player in the mammalian circadian clock system. The knockout of BMAL1 in the mouse kidney significantly reduced the expression level of mOat3 and decreased the kidney secretion of anionic drugs [65]. Indoxyl sulfate, a metabolite and uremic toxin, induced the expression of OAT1 through both the aryl hydrocarbon receptor and epidermal growth factor receptor signaling pathways in cultured kidney cells [26]. Interestingly, estrogen receptor α increased the transcription of OAT1 in cell culture in an indirect way via the activation of two transcription factors (CBF and HNRNPK) binding to the OAT1 promoter [66]. In a rat model of vancomycin-induced nephrotoxicity, rhein treatment induced the expression of erythroid 2-related factor 2 (Nrf2) to up-regulate the expression of rat Oat1 and Oat3, leading to the relief of nephrotoxicity caused by vancomycin [19]. Another rat study on cisplatin-induced acute kidney injury revealed that hyperoside increased rat Oat1 mRNA and protein expression through regulating HNF-1α and pregnane X receptor (PXR) [20]. The OAT2 gene was significantly up-regulated by the direct binding of HNF-4α. Bile acids reduced OAT2 gene expression by inhibiting the expression and transcriptional activity of HNF-4α [67]. All-trans retinoic acid repressed the expression of human OAT2, as well as other essential liver transporters, via the activation of a retinoid X receptor (RXR)/retinoic acid receptor (RAR) heterodimer in cultured liver cells [68]. In addition, the liver receptor homolog-1 (LRH-1) positively regulated the mRNA and protein expression of OAT2 in vitro and in vivo via direct binding to the OAT2 promotor [69]. A recent article showed that staphylococcal nuclease and Tudor domain-containing 1 (SND1) was bound to the 3′ untranslated region of OAT2 mRNA and inhibited OAT2 translation, resulting in reduced OAT2 protein expression in hepatocellular carcinoma cells [70]. The other OATs in the liver, OAT5 and OAT7, were found to be transactivated and modulated by HNF-1α, as well as HNF-4α, via direct binding to their promoters [71,72]. The tissue-specific expression of the uric acid transporter URAT1 in the liver and kidney was collectively modulated by transcriptional activation by the HNF-1α/β heterodimer and transcriptional inhibition by hypermethylation in the URAT1 promoter region [73]. 27-hydroxycholesterol increased the expression of URAT1 by activating estrogen receptors and the estrogen response elements on the URAT1 gene promoter region in cell culture and mouse kidneys [74].

#### 3.1.3. Regulation by miRNAs

miRNAs are small non-coding RNA molecules capable of binding to mRNA molecules, resulting in the degradation of mRNAs or inhibition of the following protein translation process [53,55]. In human kidney cells, the expression and activity of OAT1 were up-regulated by miR-223, as well as other factors such as indoxyl sulfate and reactive oxygen species. Also, the chemical inhibition of miR-223 decreased OAT1 transport activity [26]. In human hepatocytes, the mRNA expression of OAT2 was negatively correlated with the expression of several miRNAs, such as miR-21 and miR-29a-3p [54,75,76]. In addition, chenodeoxycholic acid treatment in human hepatocytes down-regulated the expression of OAT2 mRNA. In the meantime, the expression of several miRNAs changed significantly with positive and negative correlations to the expression of OAT2 mRNA, implying possible regulation roles of those miRNAs [77]. A recent study showed that miR-34a could directly bind the promoter region of URAT1 and reduce the promoter activity in vitro, resulting in reduced expression of the transporter. Also, the expression level of Urat1 mRNA in mice was negatively correlated with the expression level of miR-34a [78]. Since miRNA expressions are highly variable across different cells and tissues, the details and mechanisms of OAT regulation by miRNA still require further investigation [56].

#### 3.1.4. mRNA Alternative Splicing

mRNA splicing is the process of modifying precursor mRNAs to form mature mRNAs. mRNA alternative splicing is the process where different splicing patterns produce multiple distinct mRNAs from one gene. Those different mRNAs can then yield multiple proteins after the translation process, which may have different amino acid sequences and potentially different functions. In humans, exon skipping is the most abundant form of mRNA alternative splicing [21,53,55]. Several mRNA transcription variants were discovered for both human OAT1 and OAT3. However, their differences and functional impacts still require further investigation [79]. As for OAT2, the OAT2-tv1 variant had normal cell surface expression in three OAT2-overexpressing cell lines, while the other variant, OAT2-tv2, was unable to move to the cell surface and displayed no transport activity toward typical OAT2 substrates [79]. Shen et al. summarized multiple OAT2 substrates, which were transported differentially by transcription variants of OAT2 resulting from mRNA alternative splicing. Known substrates of OAT2-548aa, such as para-aminohippurate, estrone-3-sulfate, glutarate, succinate, and paclitaxel, cannot be transported by OAT2-546aa, indicating different substrate specificity between the alternative splicing variants of OAT2 [80].

### 3.2. Regulation of OATs by Post-Translational Control

Post-translational regulation is a process that modifies the function of the target protein through covalently attaching various functional group(s) to the amino acid side chains of such a protein. Therefore, post-translational regulation can also be called post-translational modifications (PTMs). The majority of PTMs are reversible, and specific enzymes catalyze the addition or removal of the modification. These modifications lead to changes in the function, expression, or stability of the target proteins. PTMs add diversity to the functions of the proteins. Since different PTMs can modify different amino acids of the target proteins separately or concurrently, the functional diversities of the target proteins far surpass their molecular diversities. OATs are subjected to several PTMs, including ubiquitination, palmitoylation, SUMOylation, and phosphorylation. In this review, we will update the recent advances in identifying the enzymes, signaling pathways, and modulators that influence the PTMs of OATs.

#### 3.2.1. Regulation of OATs by Ubiquitination

**Figure 1:**
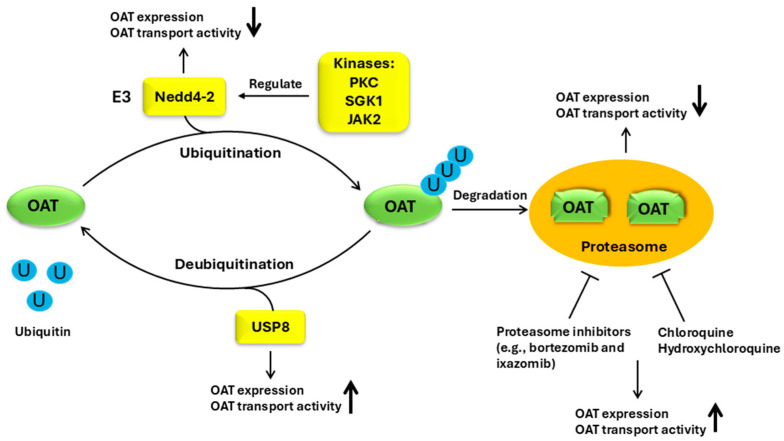
Regulation of OATs by ubiquitination. OAT, organic anion transporter; U, ubiquitin; Nedd4-2, neural precursor cell expressed developmentally down-regulated 4-2; PKC, protein kinase C; SGK1, serum- and glucocorticoid-inducible kinases 1; JAK2, janus tyrosine kinase 2; USP8, ubiquitin-specific peptidase 8.

The quantity of OATs at the cell membrane is imperative for their transport activity. Like all other transporters, OATs were thought for a long time to be immobile resident plasma membrane proteins. Our lab later discovered that members of the OAT family internalize from and recycle back to the cell surface in a constitutive manner [81]. An essential event that leads to OAT internalization is the conjugation of a ubiquitin molecule (an 8 kDa polypeptide) to the lysine residues on OATs at the cell surface, a process called ubiquitination [82]. The ubiquitination of OATs initiates the internalization of the transporter from the plasma membrane to intracellular early endosomes. Once in the early endosome, the ubiquitinated OAT can either be deubiquitinated and recycled back to the cell surface or targets to proteasome for degradation. Thus, changes in the activity of OAT-specific ubiquitin ligases and OAT-specific deubiquitinases and changes in the activity of proteasomes will likely alter OAT ubiquitination, which leads to changes in OAT trafficking (altered rates of internalization, recycling, and degradation), ultimately leading to changes in OAT abundance at the plasma membrane and OAT transport activity (Figure 1).

##### Regulation of OATs by Enzymes Involved in Ubiquitination

Ubiquitination, the conjugation of ubiquitin molecules to target proteins, is catalyzed by a series of enzymes in a consecutive order as follows: ubiquitin-activating enzyme (E1), ubiquitin-conjugating enzyme (E2), and ubiquitin ligase (E3). Nedd4-1 and Nedd4-2 are two E3 ubiquitin ligases that catalyze the last step of OAT ubiquitination. The distinction between these two ligases is that Nedd4-2-mediated OAT ubiquitination is dependent on protein kinase C (PKC), whereas Nedd4-1-mediated OAT ubiquitination is insensitive to PKC [83]. PKC activation promotes the association between Nedd4-2 and OAT, which leads to an enhanced OAT ubiquitination and subsequently an increased OAT internalization and degradation. As a result, the amount of OATs at the cell surface and OAT transport activity are both reduced [83]. Nedd4-1 and Nedd4-2 interact with OATs through their WW domains. Nedd4-1 has four WW domains. When each of the four WW domains was inactivated by mutating two amino acid residues (Mut-WW1: V210W/H212G, Mut-WW2: V367W/H369G, Mut-WW3: I440W/H442G, and Mut-WW4: I492W/H494G, respectively), only Mut-WW2 and Mut-WW3 showed a dramatical decrease in their ability to bind and ubiquitinate hOAT1 [84]. In addition to PKC, other protein kinases such as serum- and glucocorticoid-inducible kinases 1 (sgk1) [85] and janus tyrosine kinase 2 (JAK2) also regulated OATs through Nedd4-2 [86]. Countering ubiquitination is an event called deubiquitination that eliminates attached ubiquitin molecules from the target proteins, catalyzed by deubiquitinating enzymes (DUBs). Ubiquitination and deubiquitination form an opposite pair and play important roles in a variety of physiological and pathological processes [87,88,89,90]. So far, about 100 human DUBs have been identified. The study from our laboratory showed that overexpression of USP8, a DUB, in cultured cells, reduced OAT1 ubiquitination. As a result, OAT1 expression at the cell surface and transport activity were enhanced [91].

##### Regulation of OATs by Proteasome Inhibitors

Proteasomes and lysosomes are the two major systems through which cells degrade cellular proteins. We demonstrated that in contrast to most of the membrane proteins which are degraded in lysosomes, ubiquitinated OATs degrade in proteasomes [92]. A proteasome consists of two subunits, namely a catalytic core particle (called 20S proteasome) and one or two terminal 19S regulatory particle(s). The modification of proteasome activity can potentially result in altered OAT function.

Bortezomib and carfilzomib are anti-cancer drugs approved by the FDA which act as selective proteasome inhibitors for suppressing 20S proteasome activity. We discovered that the treatment of OAT1-expressing HEK293 cells with therapeutic concentrations of bortezomib or carfilzomib resulted in a remarkable increase in ubiquitinated OAT1, which was in parallel with an increase in OAT1 expression and transport activity and a decrease in the rate of OAT1 degradation. Mirrored with this, we also observed a significant decrease in 20S proteasome activity [93]. Similar results were observed in OAT3-expressing COS-7 cells treated with bortezomib and carfilzomib. The observation from cultured cells was further validated in animals. The administration of bortezomib in Sprague Dawley rats induced the augmentation of rOat3 ubiquitination and membrane expression in the rat kidneys [92]. Ixazomib, oprozomib, and delanzomib are a class of oral proteasome inhibitors for multiple myeloma treatment. An investigation of the underlying mechanism of these drugs on OAT function revealed that by suppressing 20S proteasome activity, all three drugs significantly increased the amount of ubiquitinated OAT3, which was in consistence with a decreased OAT3 degradation rate and an enhancement in OAT3 expression and transport activity [94].

We recently also discovered the novel roles of chloroquine (CQ) and hydroxychloroquine (HCQ), two well-known anti-malarial drugs, in stimulating OAT transport function by suppressing proteasome activity [95]. CQ and HCQ considerably augmented the buildup of ubiquitinated OAT3 in cultured COS-7 cells, which was in parallel with a decrease in 20S proteasomal activity and an increase in OAT3 expression and OAT3-mediated transport of estrone sulfate, a prototypical OAT3 substrate. Further mechanistic study showed that the enhanced OAT3 transport activity following CQ and HCQ treatment resulted from an increase in maximum transport velocity and a slowing down of transporter degradation. Significant amounts of CQ and HCQ are removed via kidney excretion. Their plasma concentrations in patients range from 650 to 1300 ng/mL (2.0–4.1 µM) for CQ and 1161–2436 ng/mL (3.5–7.3 µM) for HCQ [96,97]. Thus, the concentration (10 µM) utilized in our investigation in cultured cells are within the clinically therapeutic scope, which provides a better clinical understanding into functional changes in OATs in patients taking CQ and/or HCQ. Like the inhibitors specific for suppressing 20S proteasomes, several deubiquitinases are bound to the 19S regulatory particles of proteasomes [98]. It has been recently shown that the inhibition of 19S proteasome-bound deubiquitinase activity could act as an alternative approach for the treatment of cancer [99,100]. Thus, it would be an attractive idea to explore whether inhibitors for the 19S regulatory particles of proteasomes are a novel target for OAT regulation.

Drawing from the examples discussed above in this section, we unveiled a novel role of many drugs that act as proteasome inhibitors in regulating OAT function. By suppressing proteasome activity, these drugs can prevent/slow down OAT degradation, which leads to an enhancement in OAT expression and transport activity. This finding provides a new insight into regulating OAT function. Given that OATs transport numerous endogenous substrates, the drugs that act as proteasome inhibitors can enhance the renal excretion of nutrients, metabolites, signaling molecules, and other important OAT substrates, and as a result, the body homeostasis can be significantly disturbed. In addition, the inhibition of proteasomes enhances the OAT transport activity, resulting in an increased elimination of the drugs that are OAT substances and in doing so, changing the efficacy of these drugs. Finally, targeting proteasomes can be a potential approach to reverse decreased OAT expression under various pathological conditions. Although the studies described above were mainly carried out in cultured cells of kidney origin and kidney tissue, OATs are also richly expressed in the brain. Therefore, proteasomal inhibition may potentially increase the expressions and functions of OATs in the brain and other organs.

#### 3.2.2. Regulation of OATs by Palmitoylation

Palmitoylation is the covalent attachment of 16-C palmitate to one or more cysteine residues of the target proteins. Palmitoylation is a highly dynamic and reversible modification, and repeated cycles of palmitoylation/depalmitoylation play an influential role in regulating protein localization, trafficking, stability, and activity [101,102]. Palmitoylation at different sites of the target protein may control distinct functions of that protein. The palmitoylation process is catalyzed by a family of 23 mammalian palmitoyltransferases that are also known as DHHCs in reference to the conserved amino acid sequence Asp (D)-His (H)-His (H)-Cys (C) in the active site. These DHHCs differ in their tissue distribution, subcellular localization, and substrate specificity, and may regulate their target protein at multiple cellular locations such as the endoplasmic reticulum, Golgi, endosome, and plasma membrane [102]. For example, the DHHCs identified as regulators of potassium channel BK are expressed at the endoplasmic reticulum, Golgi, and cell membrane, suggesting that the BK channel may be regulated at multiple sites throughout the trafficking process to the cell membrane. Furthermore, different sites of palmitoylation within the same target protein may be regulated by distinct DHHCs. Compared to the large number of palmitoyltransferases (DHHCs), the number of the enzymes responsible for the removal of palmitate from the substrate proteins is relatively limited, including acyl-protein thioesterases (APTs) and alpha/beta hydrolase domain-containing protein 17 (ABHD17) family [102]. Palmitoylation is linked with many human diseases. For example, the functionally defective DHHC9 is related to X-linked intellectual disability and epilepsy and mutations in ZDHHC15 and ZDHHC8 are related to X-linked intellectual disability and schizophrenia, separately [103,104,105]. In the instances of pathogen infections, the palmitoylation of Cys141 and Cys498 residues on the ACE2 receptor by DHHC3 and the depalmitoylation by APT1 are critical for ACE2 to be targeted to the membrane and its release through extracellular vesicles [106]. A recent evaluation of the role of palmitoylation in kidney diseases revealed that protein palmitoylation is critical for the localization and expression of PKD1 (Polycystin 1), which plays a vital role in autosomal dominant polycystic kidney disease [107]. An abnormal level of DHHC9 is observed in rodents and humans with chronic kidney diseases [108].

**Figure 2:**
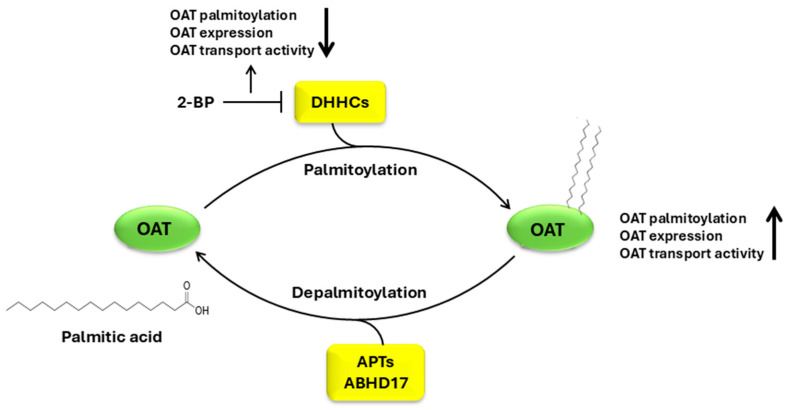
Regulation of OATs by palmitoylation. OAT, organic anion transporter; DHHC, palmitoyltransferase DHHC; APT, acyl-protein thioesterase; ABHD17, alpha/beta hydrolase domain-containing protein 17; 2-BP, 2-bromopalmitic acid.

The members of the OAT family belong to the solute carrier (SLC) superfamily of membrane transporters (~400 solute carriers). So far, palmitoylation has only been studied in a few transporters in this superfamily such as glucose transporters (GLUT1/SLC2A1 and GLUT4/SLC2A4), monoamine transporters (NET/SLC6A2, DAT/SLC6A3, and SERT/SLC6A4), the sodium–calcium exchanger (NCX1/SLC8A1), and the sodium-dependent bile acid transporter (hASBT/SLC10A2) [109]. The unpublished data from our laboratory showed that OAT1 and OAT3 are subjected to regulation by palmitoylation. Using a resin-assisted capture (RAC) assay [110], we treated OAT1-expressing cells with hydroxylamine (NH4OH) that specifically cleaved the endogenous palmitate moieties on OAT1 (Tris as negative controls), and the liberated side chains were captured by thiopropyl sepharose resin, followed by immunoblotting with an anti-OAT1 antibody to detect palmitoylated OAT1. We observed that OAT1 was palmitoylated in NH4OH-treated samples, whereas Tris-treated controls displayed negligible signals, demonstrating the specificity of OAT1 palmitoylation. Preincubating OAT1-expressing cells with 2-bromopalmitic acid (2-BP), a general blocker for palmitoylation, resulted in a significant decrease in OAT1 palmitoylation, which correlated well with a decrease in OAT1 expression and OAT1-mediated transport of para-aminohippuric acid, a prototypic substrate for OAT1, suggesting that palmitoylation affects OAT1 function. Similar to OAT1, OAT3 is also subjected to regulation by palmitoylation. Interestingly, OAT1 and OAT3 seem to be catalyzed by different sets of palmitoyltransferases (DHHCs). Even though OAT1 and OAT3 share a common feature in that they both recognize a wide range of substrates, they are different in many ways, e.g., substrate specificity, expression, and regulation [2]. For instance, based on metabolomics data obtained from Oat1 and Oat3 knockout mice, they each possesses distinct metabolites as preferred substrates, and OAT3 is more likely to interact with more complicated molecules with more rings and chiral centers [8]. Therefore, the identification of palmitoyltransferases (DHHCs) that are specific to each OAT isoform is essential to fully establish the regulations of OAT1 or OAT3 (Figure 2).

Despite the critical role palmitoylation plays in numerous physiological events such as membrane transport, signal transduction, cell growth and development, immune response, and other key processes, there remains a lack of inhibitors specific to the enzymes involved in the palmitoylation, and even fewer have entered clinical trials. One of the primary challenges is that limited information on crystal structures of most enzymes is available. Overcoming these obstacles will be vitally important for the development of effective inhibitors targeting specific enzymes for palmitoylation.

Currently, there is no common consensus sequence for protein palmitoylation. However, the known palmitoylated cysteine residues do have certain shared characteristics as follows: 1. the nearby amino acids are often basic or hydrophobic and 2. the palmitoylation sites are usually located in the cytoplasmic regions next to transmembrane domains or within the transmembrane domains. Several online software programs such as MDD-Palm (http://csb.cse.yzu.edu.tw/MDDPalm/, accessed on 21 October 2024) and GPS-Palm (http://gpspalm.biocuckoo.cn/, accessed on 21 October 2024) can be used to predict the potential palmitoylation sites of OAT1 and OAT3 [111,112]. Nevertheless, some palmitoylation sites can in fact reside outside the consensus motif. Therefore, combined experimental approaches of mass spectrometry analysis and site-directed mutagenesis are needed to map the precise sites for OAT palmitoylation.

The underlying mechanism of how palmitoylation affects OAT function requires in-depth investigation. As OATs undergo constitutive trafficking among different cellular organelles, namely the plasma membrane, intracellular endosomes, and proteasomes, palmitoylation can alter any steps of OAT trafficking (rates of internalization, recycling, and degradation) and therefore alter OAT abundance at the plasma membrane and OAT transport activity. Furthermore, we previously demonstrated through chemical crosslinking and gel filtration chromatography experiments that the functional OAT forms a trimer and higher order of oligomers [113]. Whether palmitoylation affects OATs to form multimeric complexes is another question to be addressed.

#### 3.2.3. Regulation of OATs by SUMOylation

SUMOylation is the covalent attachment of SUMO1, SUMO2, or SUMO3, polypeptides of ~12 kDa, either as monomers or as chains of oligomers, to the target proteins and is another type of post-translational modification known to be essential in numerous biological events, including protein stability, transcription control, cellular reaction to stress, and cell apoptosis. Irregular SUMOylation has been connected to various pathological conditions, such as neurodegenerative diseases, diabetes, cardiovascular disorders, and malignant tumors [114,115,116]. SUMOylation involves three enzymes (E1, E2, and E3) in a chain of events. The first step is the activation of SUMO molecules by SUMO-activating enzyme E1. The activated SUMO molecules are then transferred to SUMO-conjugating enzyme E2, also called ubiquitin-conjugating enzyme 9 (Ubc9). During this process, a thioester bond is created connecting the activated SUMO and Ubc9. Ubc9 then teams up with SUMO-ligating enzyme E3, conjugating SUMO to a lysine residue of a target protein. Our laboratory established that OATs are subjected to modification by SUMOylation, specifically modification by SUMO2 and SUMO3 but not by SUMO1 [117]. We discovered that under basal conditions (without any stimuli/treatment), OATs are SUMOylated both in cell culture and rat kidneys and exhibit tranport activity [118,119,120]. An augmentation in OAT SUMOylation resulting from stimuli will lead to an increase in OAT expression and transport activity [117,118,119,120]. Our recent work showed that ginkgolic acid (GA), by interacting with the SUMO-activating enzyme E1 and thus hindering the SUMO activation step, inhibited OAT3 SUMOylation, expression, and transport activity [121]. GA is one of the main components in the extract of Ginkgo biloba leaves that have long been utilized in traditional Chinese medicine and as a food supplement for relieving dementia and high blood pressure, as well as in stroke recovery. In addition to GA, other products from natural resources that have inhibitory effect on SUMO-activating enzyme E1 are anacardic acid, kerriamycin B, and tannic acid [122,123,124]. In particular, a drug candidate (TAK-981) developed to target SUMO-activating enzyme E1 is undergoing clinical trials for treatment of multiple blood cancers and solid tumors [125,126,127]. This is the first drug candidate targeting specific SUMOylation enzymes to enter the clinical stage. IV-administering TAK-981 in mice with colorectal and lymphoma xenograft tumors significantly decreased the levels of SUMOylated proteins and shrank the size of the tumor [125,126,127]. Other natural products such as spectomycin B1, chaetochromin A, and viomellein have also been found to have inhibitory effects on ubiquitin-conjugating enzyme 9 (Ubc9) [122,124,128].

**Figure 3:**
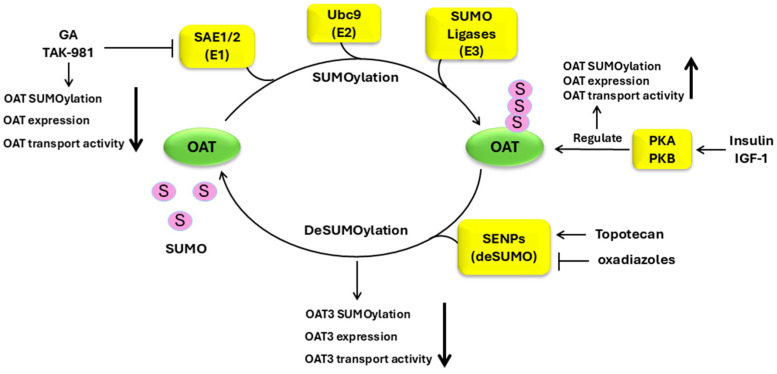
Regulation of OATs by SUMOylation. OAT, organic anion transporter; S, SUMO; SAE1/2, SUMO-activating enzyme E1/2; Ubc9, ubiquitin-conjugating enzyme 9; SENP, sentrin-specific protease; GA, ginkgolic acid; PKA, protein kinase A; PKB, protein kinase B; IGF-1, insulin-like growth factor 1.

SUMOylation is dynamic and reversible, and the proteases that remove SUMO from target proteins consist of ubiquitin-like specific protease (Ulp) in yeast and the members of the SENP family in mammals [129,130,131,132]. An alteration in the expression of SENPs has been linked to various pathophysiological conditions, such as bladder cancer, hepatocellular carcinoma, congenital heart defects, and cardiac dysfunction [133,134,135]. Our laboratory demonstrated that the overexpression of SENP2, a member of the SENP family, in COS-7 cells resulted in a reduced OAT3 SUMOylation, protein expression, and transport activity [136]. Moreover, depletion of the endogenous SENP2 with SENP2-specific siRNA resulted in an augmented OAT3 SUMOylation, protein expression, and transport activity. SENP2 exerted its effect by directly interacting with OAT3 both in cell culture and in rodent kidneys [136]. Our recent work showed that topotecan, by enhancing the interaction between OAT3 and SENP2, magnified the de-SUMOylation of OAT3 and as a result, accelerated OAT3 degradation, which resulted in a reduced OAT3 expression and transport activity [121]. Topotecan is a semi-synthetic derivative of camptothecin, a compound isolated from the Chinese yew tree, Camptotheca acuminate. The US FDA has approved topotecan for the treatment of cervical, ovarian, and small-cell lung cancer [137,138,139] through its inhibitory action on DNA topoisomerase I [140,141,142,143,144]. Topotecan has also been shown to reduce the levels of protein SUMOylation in human glioblastoma multiforme, thereby causing remarkable alterations in the cell cycle and cell metabolism [140,141]. 1,2,5-Oxadiazoles were created as a group of SENP2 inhibitors, which might have therapeutic potential for many diseases [145]. Therefore, the enzymes that either promote or demote OAT SUMOylation can be potential targets for regulating OAT function. The SUMOylation of OATs is also under the control of the protein kinase A signaling pathway. PKA activation enhanced the conjugation of SUMO2/3 to OATs, and such an increase can be abrogated by PKA-specific inhibitor H-89 [117,118,119] (Figure 3). A more direct evidence that SUMOylation mediates the PKA effects on OAT function can be gained by generating mutated OATs, in which specific SUMOylation site(s) are mutated.

Protein SUMOylation often occurs at a consensus motif, Ψ-K-X-D/E (Ψ is a hydrophobic amino acid, K is the lysine residue for SUMO conjugation, X is any amino acid, D is an aspartic acid, and E is a glutamic acid). An online software called SUMOplot™ (https://www.abcepta.com/sumoplot, accessed on 21 October 2024) (Abcepta, San Diego, CA, USA) is used to predict several potential SUMOylation sites within the sequences of OAT1 and OAT3. Confirming these sites through site-directed mutagenesis and investigating the relationship of the SUMOylation sites and OAT transport activity will be an interesting direction for future investigations.

#### 3.2.4. Regulation of OATs by Phosphorylation

Phosphorylation is an important PTM which attaches negatively charged phosphoryl group(s) to serine, threonine, or tyrosine residues of the target proteins. The phosphorylation process is catalyzed by protein kinases. The conjugation of the phosphoryl group to the target proteins causes changes in protein conformation, protein activity, protein stability, cellular localization of the protein, or protein–protein interaction. The regulation of OATs by phosphorylation can happen in a direct or indirect manner. The direct manner involves the phosphorylation of the OAT itself, whereas the indirect manner involves the phosphorylation of OAT-interacting proteins rather than the OAT itself. The best example of direct OAT phosphorylation came from our study, where the treatment of OAT3-expressing cells with protein kinase A (PKA) activator Bt2-cAMP increased OAT3 phosphorylation significantly, which paralleled with an augmented OAT3 transport activity. Furthermore, the treatment of OAT3-expressing cells with insulin-like growth factor 1 (IGF-1), an upstream hormonal activator of PKA, enhanced OAT3 phosphorylation, which correlated well with and enhanced OAT3 protein expression and transport activity. The stimulatory effect of IGF-1 was blocked by H-89 (a selective PKA inhibitor). Thus, the IGF-1/PKA signaling pathway up-regulated OAT3 expression and transport activity via the direct phosphorylation of OAT3 [146]. More direct evidence that phosphorylation mediates PKA effects on OAT function can be obtained by generating OAT mutants in which specific phosphorylation site(s) are mutated. The best examples of the regulation of OATs by indirect phosphorylation came from our study with Nedd4-2, a ubiquitin ligase. Through its binding to the OAT, Nedd4-2 catalyzes OAT ubiquitination, which leads to OAT internalization from the cell surface and subsequent degradation in proteasomes. As a result, OAT expression and transport activity are reduced. In contrast to the direct phosphorylation of OATs, many protein kinases, hormones, and chemicals regulate OATs through phosphorylating Nedd4-2 at different sites, which causes distinct conformational change in Nedd4-2, either strengthening or weakening the binding of Nedd4-2 to the OAT, thereby either inhibiting or stimulating OAT transport activity.

Our published work showed that the activation of PKC reduced OAT expression and transport activity by phosphorylating Nedd4-2 rather than phosphorylating the OAT directly in cell culture [147]. The phosphorylation of Nedd4-2 by PKC resulted in an enhanced binding of Nedd4-2 to the OAT, which led to an enhanced OAT ubiquitination and a subsequent decrease in OAT expression and transport activity. A liquid chromatography tandem mass spectrometry (LC-MS/MS) analysis identified four amino acid residues on Nedd4-2, with the most significant changes in phosphorylation in response to the activation of PKC (Threonine-197, Serine-221, Serine-354, and Serine-420) [147]. Although the mutation of each of these phosphorylation sites individually did not abolish the response of these mutants to PKC activation, the mutation of these sites simultaneously eliminated the response of the quadruple mutant to PKC activation almost entirely. One of the plausible explanations is that when one site of phosphorylation is mutated, other sites may have enhanced phosphorylation to compensate for the loss of phosphorylation at the mutated site and thus, the overall phosphorylation of each single mutant would remain similar as compared to that of wild type Nedd4-2. Therefore, a concerted action of the four individual sites of phosphorylation together is required in the response of Nedd4-2 to PKC for the regulation of OAT function. Angiotensin II and the parathyroid hormone inhibited OAT transport activity through the activation of the PKC/Nedd4-2 pathway [148].

**Figure 4:**
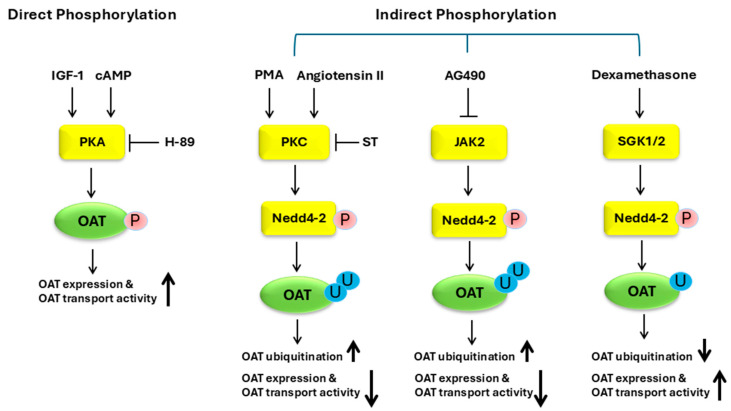
Regulation of OATs by phosphorylation. OAT, organic anion transporter; P, phosphorylation; U, ubiquitin; IGF-1, insulin-like growth factor 1; cAMP, cyclic adenosine monophosphate; PKA, protein kinase A; PKC, protein kinase C; Nedd4-2, neural precursor cell expressed developmentally down-regulated 4-2; PMA, phorbol 12-myristate 13-acetate; ST, staurosporine; JAK2, janus tyrosine kinase 2; SGK, serum- and glucocorticoid-inducible kinase.

In contrast to the inhibitory effect of PKC on OATs by phosphorylating Nedd4-2 at threonine-197, Serine-221, Serine-354, and Serine-420, other protein kinases stimulate OAT transport activity by phosphorylating Nedd4-2 at distinct sites. Serum and glucocorticoid-regulated kinase 1 (Sgk1) and serum and glucocorticoid-regulated kinase 2 (Sgk2) enhanced the transport activity of OAT3, OAT1, and OAT4 in cultured cells by phosphorylating Nedd4-2 on Ser327, which impaired the binding between OATs and Nedd4-2, thereby reducing OAT ubiquitination and enhancing OAT expression and transport activity [85,120]. Dexamethasone and insulin, upstream hormones of Sgk1 and Sgk2, enhanced OAT expression and transport activity by phosphorylating Nedd4-2 on Ser327 in cultured cells [120,149]. The phosphorylation of Nedd4-2 occurs not only on serine and threonine residues, but also on tyrosine residues. AG490, a janus tyrosine kinase 2 (JAK2) inhibitor, inhibited OAT3 protein expression and transport activity in cell culture. The decreased transport activity was a result from an augmented OAT3 ubiquitination, following a decreased tyrosine phosphorylation of Nedd4-2 and an increased binding between OAT3 and Nedd4-2. The inhibition effect on OAT3 by AG490 was abolished by knocking down the endogenous Nedd4-2 using Nedd4-2-specific siRNA [86]. Therefore, the varied regulations of OATs by various protein kinases (negative regulation vs. positive regulation) are exerted through the dynamic phosphorylation at various sites on Nedd4-2, a central convergence point/switch, thereby inducing distinct conformational changes in Nedd4-2 and altering its binding to OATs (association/dissociation), which results in a change in OAT ubiquitination, trafficking, degradation, expression, and transport activity (Figure 4).

### 3.3. Crosstalk Between Various PTMs

We now have evidence that OATs undergo different types of PTMs, including ubiquitination, palmitoylation and SUMOylation, and phosphorylation. The locations/regions and specific amino acid residues on OATs to which PTMs occur may affect different functional outcomes. Furthermore, there can be crosstalk between different PTMs or between different signaling pathways that control the PTM. For example, a single residue (serine, threonine, or tyrosine) can be phosphorylated by various protein kinases and de-phosphorylated by different phosphatases to generate entirely different functional consequences. Lysine residues can be ubiquitinated or SUMOylated. One scenario is that ubiquitin and SUMO conjugate to the same lysine residue(s) in a target protein in a competitive mode. Alternatively, ubiquitin and SUMO may conjugate to different lysine residues in a substrate protein. In such a situation, SUMO conjugation may possibly conceal a nearby site for ubiquitin conjugation. In both situations, SUMOylation may prevent the ubiquitin-mediated degradation of target proteins [150]. Our laboratory demonstrated that the activation of PKC augmented OAT3 SUMOylation at the expense of OAT3 ubiquitination in cultured cells. Thus, SUMOylation and ubiquitination may work in concert in the regulation of OAT3 via crosstalk [117]. In addition to SUMOylation, our unpublished results showed that OAT palmitoylation may also antagonize with OAT ubiquitination. Treatment of OAT-expressing cells with 2-BP, a general palmitoylation inhibitor, significantly decreased OAT palmitoylation, which is in parallel with an increase in OAT ubiquitination. Therefore, palmitoylation and ubiquitination may counteract with each other in regulating OAT trafficking and function. It would be interesting to explore whether there is interplay between OAT phosphorylation and OAT SUMOylation and between OAT phosphorylation and palmitoylation.

## 4. Conclusions

Different OAT isoforms, expressed widely in multiple organs, exhibit certain levels of substrate overlap. These OATs can undergo regulations at multiple levels such as transcriptional, translational, and post-translational modifications by signaling molecules secreted from remote tissues/organs. The novel concept/hypothesis of remote sensing and signaling describes the complicated communication network of OATs, which can be found in several excellent publications [1,151,152,153]. It is through this network of communication that OATs contribute significantly to various processes involved in physiology, pathology, and drug disposition.

## Data Availability

The original contributions presented in the study are included in the article; further inquiries can be directed to the corresponding author.
